# Redefining prognostic factors for breast cancer: YB-1 is a stronger predictor of relapse and disease-specific survival than estrogen receptor or HER-2 across all tumor subtypes

**DOI:** 10.1186/bcr2156

**Published:** 2008-10-16

**Authors:** Golareh Habibi, Samuel Leung, Jennifer H Law, Karen Gelmon, Hamid Masoudi, Dmitry Turbin, Michael Pollak, Torsten O Nielsen, David Huntsman, Sandra E Dunn

**Affiliations:** 1 Laboratory for Oncogenomic Research, Departments of Pediatrics and Experimental Medicine, Child and Family Research Institute, W. 28th Avenue, University of British Columbia, Vancouver, BC, V5Z 4H4, Canada; 2 Genetic Pathology Evaluation Center, Jack Bell Research Center, Oak Street, Vancouver, BC, V6H 3Z6, Canada; 3 Division of Medical Oncology, British Columbia Cancer Agency, West 10th Avenue, Vancouver, BC, V5Z 1L3 Canada; 4 Department of Pathology and Laboratory, West 10th Avenue, Vancouver, BC, V5Z 1L3, Canada; 5 Division of Medical Oncology, Jewish General Hospital – Lady Davis Institute, Côte Ste Catherine Road, Montreal, Quebec H3T 1E2, Canada

## Abstract

**Introduction:**

Gene expression analysis is used to subtype breast cancers such that the most aggressive tumors are identified, but translating this into clinical practice can be cumbersome. Our goal is to develop a universal biomarker that distinguishes patients at high risk across all breast cancer subtypes. We previously reported that Y-box binding protein-1 (YB-1), a transcription/translation factor, was a marker of poor prognosis in a cohort of 490 patients with breast cancer, but the study was not large enough to subtype the cancers. We therefore investigated whether YB-1 identifies patients at risk for either reduced relapse free survival or decreased r breast cancer specific survival (BCSS) across all tumor subtypes by evaluating 4,049 cases.

**Methods:**

Tumor tissue microarrays, representing 4,049 cases of invasive breast cancers with 20 years of follow up, were subtyped by the expression profiles of estrogen receptor, progesterone receptor, or HER-2. We then addressed whether YB-1 expression identified patients at higher risk for relapse and/or lower BCSS.

**Results:**

We found YB-1 to be a highly predictive biomarker of relapse (*P *< 2.5 × 10^-20^) and poor survival (*P *< 7.3 × 10^-26^) in the entire cohort and across all breast cancer subtypes. Patients with node-positive or node-negative cancer were more likely to die from the disease if YB-1 was expressed. This was further substantiated using a Cox regression model, which revealed that it was significantly associated with relapse and poor survival in a subtype independent manner (relapse patients, hazard ratio = 1.28, *P *< 8 × 10^-3^; all patients, hazard ratio = 1.45, *P *< 6.7 × 10^-7^). Moreover, YB-1 was superior to estrogen receptor and HER-2 as a prognostic marker for relapse and survival. For a subset of patients who were originally considered low risk and were therefore not given chemotherapy, YB-1 was indicative of poor survival (*P *< 7.1 × 10 ^-17^). Likewise, YB-1 was predictive of decreased BCSS in tamoxifen-treated patients (*P *= 0.001); in this setting a Cox regression model once again demonstrated it to be an independent biomarker indicating poor survival (hazard ratio = 1.70, *P *= 0.022).

**Conclusions:**

Expression of YB-1 universally identifies patients at high risk across all breast cancer subtypes and in situations where more aggressive treatment may be needed. We therefore propose that YB-1 may re-define high-risk breast cancer and thereby create opportunities for individualized therapy.

## Introduction

The overall goal of predictive oncology is to refine treatment options for patients such that they may receive optimal care without experiencing unnecessary side effects. One of the greatest challenges is the identification and implementation of biomarkers for cancer [1]. Although the antigen Ki67 is reportedly associated with poor survival it is no longer recommended as a biomarker in prognostic groups, according to a recent report from the American Society for Clinical Oncology [2]. Furthermore, this touted biomarker was disappointingly notpredictive of response to adjuvant chemoendocrine therapy in a study of about 2,000 patients enrolled in two randomized International Breast Cancer Study Group trials [3].

Many reports have shown that human epidermal growth factor receptor (HER)-2 is a marker of poor prognosis in breast cancer, following its initial report in 1987 by Slamon and coworkers [4]. This subsequently led to the development of targeted agents against it. However, where it is most clinically useful is as a predictive marker used to guide treatment decisions about whether to use agents that target this receptor such as trastuzumab [3]. In fact, HER-2 is no longer recommended as a prognostic factor in breast cancer [3]. In contrast to HER-2, the estrogen receptor (ER) is associated with good prognosis [5,6]. ER is similar to HER-2 in that many therapies targeted against it have been developed. As such, ER is more routinely used as a biomarker to guide treatment decisions about whether hormone therapy is appropriate [3]. Although HER-2 and ER have been very informative for our understanding of patient survival, and this has lead to the eventual development of targeted agents, they do not apply to all tumor typesGiven the limited number of robust biomarkers that predict poor overall survival, the question of which should be used to guide patient care remains open [7]. Breast cancers have been subdivided into four subtypes, namely luminal A, luminal B, HER-2, and basal-like (also called triple negative), based on gene expression signature [8]. The latter two types are typically more aggressive than the former two. It should be noted that a biomarker denoting poor survival across subtypes is yet to be identified.

Y-box binding protein-1 (YB-1) is a transcription and translation factor that can promote tumor growth and chemotherapy resistance by inducing growth-promoting genes such as *HER-2 *and *EGFR *(epidermal grwoth factor receptor) [9], *PCNA *(proliferating cell nuclear antigen) [10], *MDR-1*/*ABCA1 *(multi-drug resistance-1) [11], *cyclin A *[12], and *cyclin B *[12]. YB-1 (also known as nuclease sensitive protein-1 or NSEP-1) was reportedly expressed in a high-risk group of patients with *BRCA1 *mutations by cDNA microarray [13]. Perou and Sorlie also identified it by microarray analysis [8], but at the transcript level YB-1 clustered into a unique group of genes, the significance of which awaits further description. Perhaps this signature represents genes that are commonly over-expressed in breast cancer but do not to fall into a given subtype. With regard to tumor progression, targeted expression of YB-1 in the mammary gland under the WAP promoter leads to the development of tumors; 100% of mice develop invasive carcinomas within 52 months [14].

These studies indicate that YB-1 is indeed an oncogene that is important in the genesis of the disease [14]. In models of human breast cancer, inhibition of YB-1 with a dominant negative mutant (interferes with the DNA binding activity of the protein [S102A]) slows tumor cell growth [15], and this is associated with the downregulation of EGFR and HER-2 [9]. It thus appears that YB-1 is important in the development of mammary tumors and that human breast cancer cells not only express this oncoprotein but also continue to depend upon it for sustained growth and survival.

It is unclear whether YB-1 carries prognostic value for specific types of breast cancer or whether it may be a useful biomarker across all types. In a pilot study, we reported that YB-1 expression is associated with poor overall survival in a group of 490 patients with invasive breast cancer [9]. In that cohort we also found that YB-1 was expressed in more than 70% of basal-like breast cancers [16], but these studies were limited by inadequate size to determine patient survival within individual subtypes. We also lacked the power to evaluate the important question of whether YB-1 expression is associated with higher rates of relapse. To address these issues, we screened a large tissue microarray (TMA) series consisting of 4,049 invasive breast cancers with 20 years of clinical follow up. The potential value of YB-1 as a biomarker for aggressive disease was also examined in a subset of women who were originally considered to be at low risk and therefore did not receive chemotherapy. Finally, we examined the possibility that YB-1 may be associated with more aggressive disease in an adjuvant setting in which tamoxifen was given for 5 years.

## Materials and methods

The study cohort and construction of the TMAs used in the present study has previously been described [5,17]. Sections from were cut at 4 μm and immunostained with a rabbit polyclonal anti-YB-1 antibody (1:1,400; a gift from Dr Colleen Nelson, University of British Columbia, Vancouver, BC, Canada). Of note, this antibody detects marked YB-1 expression in the cytoplasm of formalin-fixed paraffin-embedded tissues, but identification of YB-1 in the nucleus is much lower than one would expect. We believe that this is because the carboxyl-terminal epitope is somehow masked during fixation, as we previously reported [9].

The immunohistochemical staining was performed using an Automated Secondary System (cat #760-4205; Ventana, Tucson, AZ, USA). Slides were stained concurrently for ER, HER-2, and progesterone receptor (PR) using standard immunoperoxidase techniques, as described previously [5,17]. Breast cancer subtypes were determined using immunohistochemical markers (HER-2, ER, and PR) to define each type. Subtype definitions were as follows: luminal A (ER-positive and/or PR positive, HER-2 negative), luminal B (ER positive and/or PR positive, HER-2 positive), triple negative (ER negative, PR negative, HER-2 negative) [18], HER-2 positive (ER negative, PR negative, HER-2 positive), and unassigned (missing data on any of the three markers). TMAs were evaluated independently by two pathologists (Drs Hamid Masoudi and Dimithry Turbin) to quantify the percentage of tumor cells staining positive for YB-1, ER, and PR as follows: negative (<1%), positive 1+ (1% to 25%), positive 2+ (25% to 75%), or positive 3+ (>75%). For the analyses, YB-1 expression was dichotomized into 0 or 1 (where 0 = negative or very low, and 1 = moderate to high [2+ and 3+]). HER-2 was considered positive if it was 3+, as we previously described [9]. Of the 4,049 samples, for 3,097 YB-1 could be scored and breast cancer specific survival (BCSS) outcome data were available for these. In addition, in 1,201 cases that could be scored for YB-1, data were available regarding relapse free survival (RFS). Immunohistochemical data was collected for samples that were blinded as to clinical outcomes.

Statistical analyses were performed using SPSS software version 13.0 (SPSS Inc., Chicago, IL, USA) and were described previously [5]. Briefly, for univariate analyses, BCSS and RFS were estimated using Kaplan-Meier curves. Differences in survival were determined using Breslow tests. For BCSS, survival time was censored at death if the cause was not breast cancer or if the patient was still alive at the end of the study. Six patients with unknown cause of death were excluded from the BCSS analysis. In addition, RFS was also censored at death if the cause was not breast cancer and if the patients were alive without any relapse at the end the study. For multivariate analyses, Cox proportional hazards models were used to calculate adjusted hazard ratios (HRs), accounting for independent covariates. A likelihood ratio was used to determine the extent of association of YB-1 expression with particular subtypes. Spearman's correlation was also used to determine the extent of correlation between YB-1 expression and other markers such as ER.

## Results and discussion

**Figure 1:**
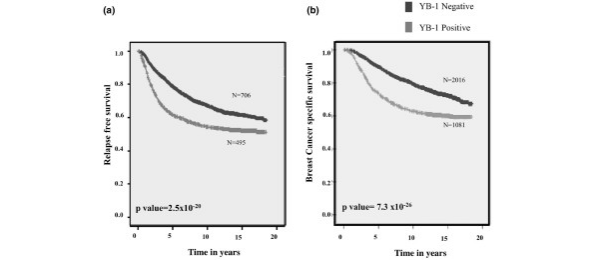
YB-1 expression is associated with relapse and poor survival in the entire cohort. **(a) **Out of a total of 1,201 patients, YB-1 was expressed in 41% (495/1201) with recurrent breast cancer. YB-1 expression was associated with shorter relapse free survival. 'N' represents the number of patients who had breast cancer recurrence (number of events). **(b) **The expression of YB-1 is associated with a significant increase in breast cancer specific deaths (BCSS). YB-1 was expressed in 35% (1,081/3,097) of patients with reduced BCSS. Patient survival was based on deaths specifically related to breast cancer and not other causes. BCSS = breast cancer specific survival; YB-1 = Y-box binding protein-1.

YB-1 was detected by immunostaining in 41% (1,644/4,049) of the patient samples, in which it was highly predictive of decreased RFS (*P *< 2.5 × 10^-20^). We observed that 80% of patients who expressed little or no YB-1 were relapse free over 5 years, as compared to 60% if the protein was highly expressed (Figure 1a). Consistent with this observation, YB-1 was associated with poor overall BCSS (*P *< 7.3 × 10^-26^; Figure 1b). Among patents expressing low levels of YB-1, about 90% were alive after 5 years, as compared with only 75% of those who expressed YB-1 (Figure 1b). Furthermore, this was the case whether patients were node positive or node negative (Additional file 1). Specifically, YB-1 was expressed in 33% of node-negative breast cancer patients, in whom it was associated with poor survival (*P *= 1.0 × 10^-13^). It was similarly expressed in 37% of node-positive patients, in whom it once again predicted poor survival (*P *= 3.6 × 10^-14^).

Of note, patients whose tumors expressed YB-1 tended to be younger (*r *= -0.203, *P *= 6.512 × 10^-17^), their tumors were of higher grade (*r *= 0.268, *P *= 2.957 × 10^-27^), and many were ER negative (*r *= -0.343, *P *= 5.452 × 10^-47^; Additional file 2). There was also a positive correlation with tumors harboring amplified HER-2 (*r *= 0.217, *P *= 8.588 × 10^-19^; Additional file 2) corroborating smaller reports by us [9] and others [19], in which cohorts of 490 and 73 patients were evaluated, respectively.

**Figure 2:**
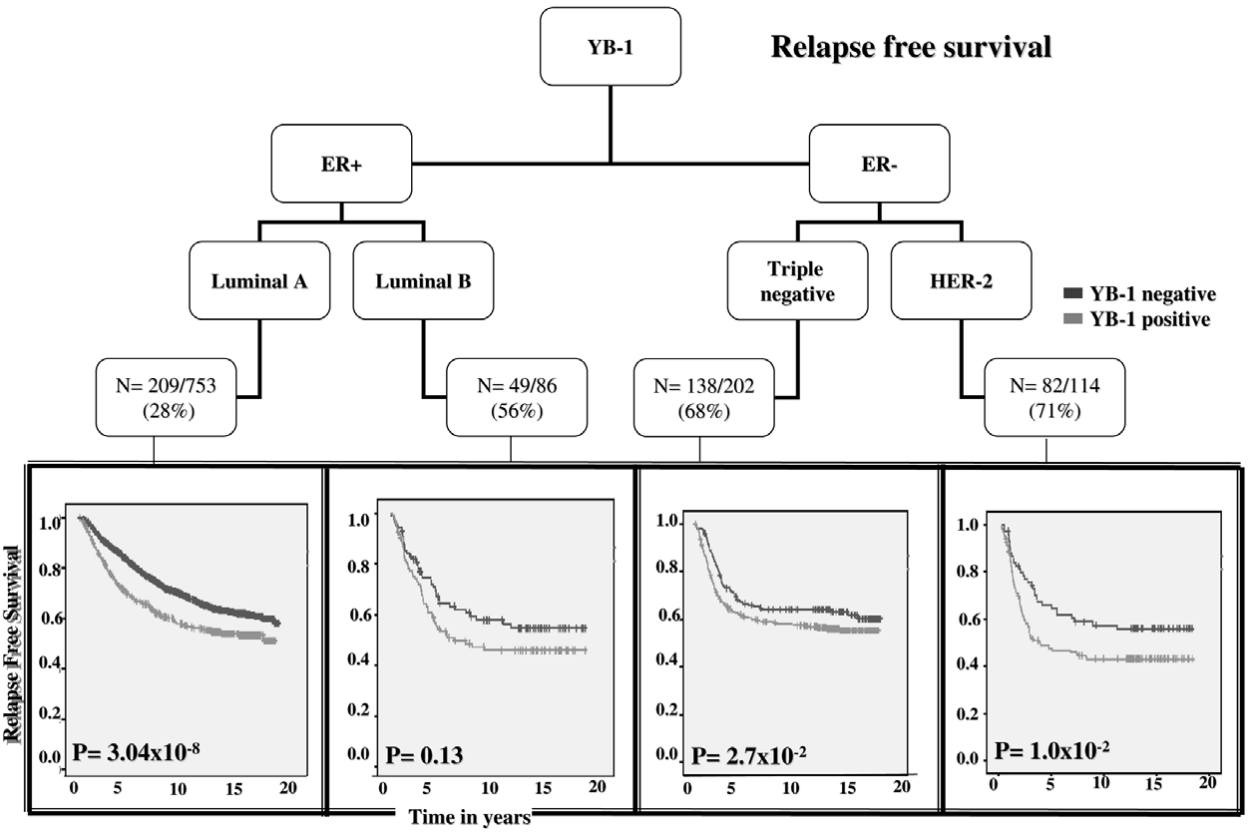
YB-1 is linked to relapse in all subtypes of breast cancer defined by hormone receptors and HER-2 status. Tumor tissue microarrays were stained for YB-1, ER, PR, and HER-2 (*n* = 2,978 cases in total for all markers). The relative distribution of YB-1 was evaluated in breast cancer subtypes. In all subtypes, the expression of YB-1 was associated with shorter relapse free survival. 'N' represents the number of YB-1 positive patients who had breast cancer relapse (number of events) in each subtype. ER, estrogen receptor; HER, human epidermal growth factor receptor; PR, progesterone receptor; YB-1, Y-box binding protein-1.

**Figure 3:**
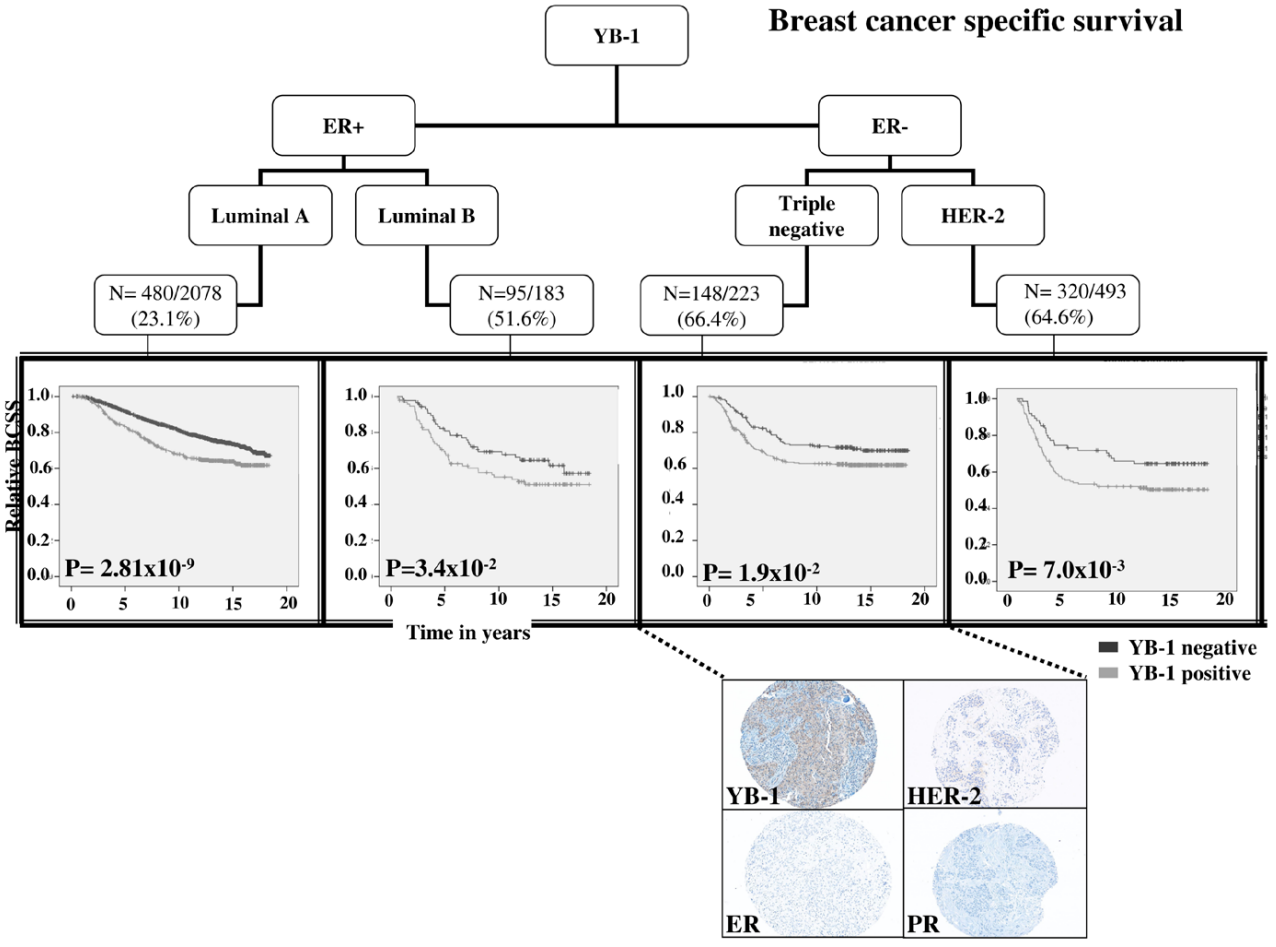
YB-1 is associated with BCSS in all subtypes of breast cancer. YB-1 is expressed in 23.1% (480/2078) of luminal A and 51.6% (95/183) of luminal B, and is more likely to be expressed in the ER-negative breast cancers, in which it was found in 64.6% (320/493) and 66.4% (148/223) of TNP and HER-2 subtypes, respectively. Its expression is associated with poor survival in all subtypes of breast cancer. 'N' represent of number of YB-1 positive patients who died from breast cancer (number of events) in each subtype. Examples of the immunostaining are represented in the inset (magnification 200×). BCSS, breast cancer specific survival; ER, estrogen receptor; YB-1, Y-box binding protein-1.

Importantly, YB-1 staining was consistently associated with disease recurrence, independent of breast cancer subtype as defined by ER, PR, and HER-2 expression (Figure 2: luminal A, *P *= 3.04 × 10^-8^; luminal B, *P *= 0.133; triple negative, *P *= 1.9 × 10^-2^; HER-2, *P *= 2.7 × 10^-2^). An exception was found in the luminal B cohort, and the lack of significance was possibly because there were only 86 patients in this subgroup and so the analysis was under-powered. Taking our observations further, BCSS was shorter when YB-1 was higher in all breast cancer subtypes (Figure 3: luminal A, *P *= 2.8 × 10^-9^; luminal B, *P *= 0.034; triple negative, *P *= 7 × 10^-3^; HER-2, *P *= 1.6 × 10^-2^). Of note is the observation that although YB-1 is expressed less frequently in the luminal A subtype, its expression was the most significant, probably because this constituted the largest group of patients overall. Whether YB-1 cooperates with estrogen to promote the growth of luminal A breast cancer is not known and is a question that we are actively addressing. To evaluate further the significance of YB-1 as a prognostic factor across breast cancer subtypes, we conducted Cox regression analyses and confirmed that it independently predicted RFS (HR = 1.284, 95% confidence interval [CI] = 1.068 to 1.544; *P *= 8 × 10^-3^) and BCSS (HR = 1.452, 95% CI = 1.253 to 1.682; P = 6.74 × 10^-7^; Additional files 3 and 4).

These findings prompted us to examine how YB-1 compared with established prognostic factors used to guide patient diagnosis and thus treatment. Importantly, YB-1 was more indicative of RFS (HR = 1.331, 95% CI = 1.169–1.516; *P *= 1.61 × 10^-5^) than HER-2 (HR = 1.256, 95% CI = 1.066 to 1.479; *P *= 6.0 × 10^-3^) or ER (HR = 0.942, 95% CI = 0.816 to 1.087; *P *= 4.13; Table 1). Similarly, YB-1 expression posed an even greater risk for reduced BCSS (HR = 1.456, 95% CI = 1.257 to 1.656; *P *= 5.6 × 10^-7^) than did HER-2 expression (HR = 1.259, 95% CI = 1.052 to 1.506; *P *= 1.2 × 10^-2^) or ER status (HR = 0.816, 95% CI = 0.720 to 0.991; *P *= 0.038; Table 2). Moreover, YB-1 was also better at predicting outcome than tumor grade (HR = 1.416, 95% CI = 1.215 to 1.651; *P *= 8.9 × 10^-6^) or patient age (HR = 1.160, 95% CI = 1.000 to 1.345; *P *= 5 × 10^-2^). We therefore conclude that YB-1 expression can be used to identify those patients who are likely to require more aggressive therapy, given its pronounced association with markedly reduced RFS and attenuated BCSS.

**Table 1:** In a multivariate analysis of recurrent breast cancer cases, the expression of YB-1 was better than HER-2 or ER in predicting events

Prognostic marker	Patients with relapse	
	
	HR (95% CI)	*P *value
Nodal status		
Positive versus negative	1.951 (1.725 to 2.206)	1.7 × 10^-26^

Tumor size (cm)		
2 to 5	1.392 (1.227 to 1.579)	2.8 × 10^-7^
>5	1.921 (1.227 to 2.445)	1.2 × 10^-7^

Grade		
III versus II versus I	1.300 (1.139 to 1.482)	9.6 × 10^-5^

Age (years)		
<50 versus ≥ 50	1.000 (0.878 to 1.139)	1.000

ER		
Positive versus negative	0.942 (0.816 to 1.087)	0.41

HER-2		
Positive versus negative	1.256 (1.066 to 1.479)	6.0 × 10^-3^

YB-1		
Positive versus negative	1.331 (1.169 to 1.516)	1.6 × 10^-5^

The predictive power of YB-1 was similar to that of tumor grade and smaller tumor size, but not as great as that of nodal status. CI, confidence interval; ER, estrogen receptor; HER, human epidermal growth factor receptor; HR, hazard ratio; YB-1 = Y-box binding protein-1.

**Table 2:** In a multivariate analysis of breast cancer cases, the expression of YB-1 was better than HER-2 or ER in predicting reduced BCSS

Prognostic marker	Patients with reduced BCSS	
	
	HR (95% CI)	*P *value
Nodal status		
Positive versus negative	2.405 (2.083 to 2.776)	4.6 × 10^-33^

Tumor size (cm)		
2 to 5	1.605 (1.387 to 1.859)	2.8 × 10^-10^
>5	2.269 (1.749 to 2.943)	2.8 × 10^-10^

Grade		
III versus II versus I	1.416 (1.215 to 1.651)	8.9 × 10^-6^

Age (years)		
<50 versus = 50	1.160 (1.000 to 1.345)	5.0 × 10^-2^

ER		
Positive versus negative	0.816 (0.720 to 0.991)	3.8 × 10^-2^

Her-2		
Positive versus negative	1.259 (1.052 to 1.506)	1.2 × 10^-2^

YB-1		
Positive versus negative	1.456 (1.257 to 1.656)	5.6 × 10^-7^

YB-1 was approximately equal to the power of tumor grade yet not as accurate as tumor size or nodal status in predicting reduced BCSS. BCSS, breast cancer specific survival; CI, confidence interval; ER, estrogen receptor; HER, human epidermal growth factor receptor; HR, hazard ratio; YB-1 = Y-box binding protein-1.

**Figure 4:**
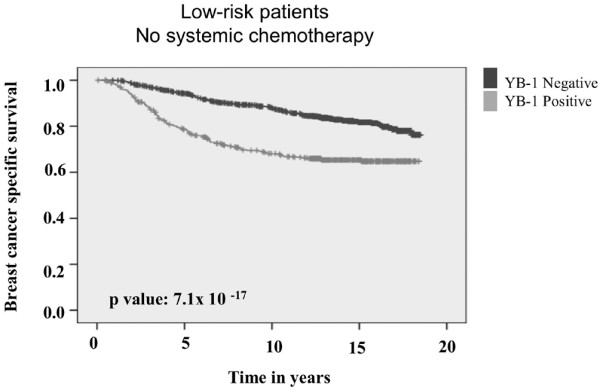
YB-1 is a high risk factor in low-risk patients. YB-1 is expressed in 34% (437/1292) of low-risk patients who received no systemic therapy. 'N' represents the number of patients who died from breast cancer (number of events) in each category. Survival was stratified based on whether tumors expressed YB-1 (light grey line) or did not (black line). YB-1, Y-box binding protein-1.

As breast cancer is being diagnosed earlier, there is a need to identify those individuals who may or may not need chemotherapy. Patients who have grade I to II tumors with no evidence for lymph node involvement (T1N0) have been categorized as being in a low-risk group, in which the odds of survival should be very good. Within the TMA we analyzed, there were 1,292 cases considered to be low risk. However, if these patients had tumors that expressed YB-1 they were more likely to die from breast cancer (Figure 4). We found that 437 out of 1,292 (34%) cases expressed YB-1, indicating that a significant proportion of patients considered to be at low risk were more likely to die from the disease than expected, suggesting that more aggressive treatment might have improved their outcome. Using 5-year survival as a benchmark, we found that more than 95% of patients were alive if YB-1 was not highly expressed. However, if YB-1 expression was high then only 75% of patients were alive at 5 years (Figure 4; *P *= 7.09 × 10^-17^). By multivariate analysis, YB-1 expression was a significant adverse prognostic biomarker in patients who did not receive chemotherapy (HR = 1.898, 95% CI = 1.460 to 2.465; *P *< 1.67 × 10^-6^; Additional file 5). Again, it was better at predicting BCSS than HER-2 or ER (Additional file 5).

**Figure 5:**
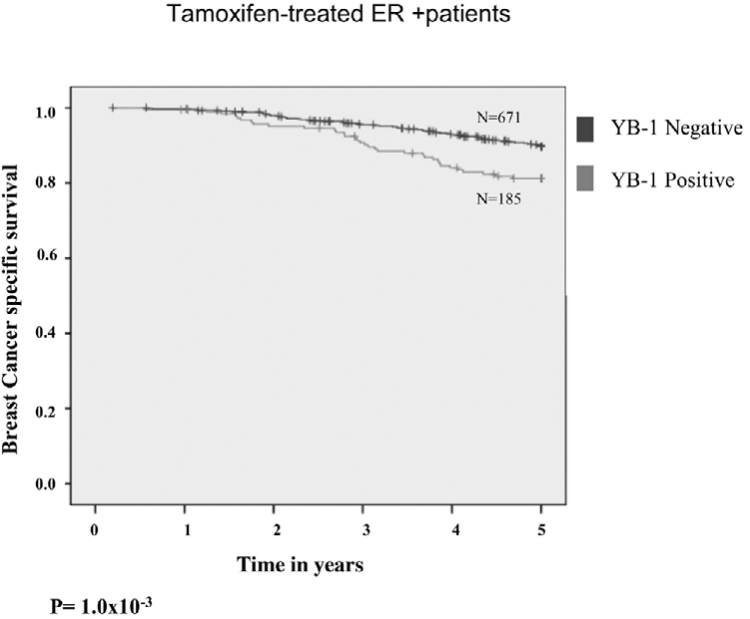
In the adjuvant setting expression of YB-1 is indicative of reduced BCSS. YB-1 expression was evaluated in a nested cohort of 671 tamoxifen-treated patients. Twenty-eight percent of patients expressed YB-1 (185/671), in whom its expression was associated with reduced BCSS. Survival was assessed over 5 years, because this is the standard duration of tamoxifen administration. ER, estrogen receptor; YB-1, Y-box binding protein-1.

To further this line of investigation, we addressed whether YB-1 emerged as a prognostic marker in patients taking tamoxifen. In a nested cohort of ER-positive breast cancer patients treated with tamoxifen (*n *= 671), YB-1 expression was associated with reduced BCSS (Figure 5). Of note, YB-1 was expressed in 185 out of 671 cases (28%), indicating that expression of this protein is not an uncommon event in these tumors. We chose to evaluate this within a 5-year time-frame, given that this is the typical duration of tamoxifen administration. In a Cox regression model, YB-1 (HR = 1.707; *P *= 2.2 × 10^-2^) was independently associated with a greater risk for breast cancer related death (Additional file 6). Thus, in the adjuvant setting, YB-1 can also serve as a useful biomarker of aggressive disease. It would therefore be interesting to evaluate YB-1 within the context of more contemporary standard-of-care hormone therapies such as raloxifene, letrozole, anastrozole, and exemestane.

Our data indicate that YB-1 is a strong prognostic marker for all subtypes of human breast cancer, even in cases that were thought to be low risk and therefore did not need chemotherapy. We also extend preliminary clinical data from others suggesting that YB-1 expression distinguish a high-risk group of breast cancer patients. Notably, Janz and coworkers [20] observed YB-1 expression in 49% (20/42) of patients receiving chemotherapy, and there was a trend toward an association of YB-1 with poor disease-free survival, although this finding did not reach statistical significance. The same authors also examined YB-1 expression in a cohort of 42 low-risk cancers not treated with chemotherapy and found that it was expressed in 76% (32/42) of those cases. Notably, none of the patients who had low YB-1 expression relapsed, whereas 30% of those with high levels did (*P *< 1.1 × 10^-2^). In addition, Huang and colleagues [21], in a study of 42 patients, reported that YB-1 was associated with recurrence after adjuvant chemotherapy, although long-term outcomes were not evaluated. We now provide definitive retrospective data that indicate that YB-1 expression in low-risk patients is strongly associated with a higher rate of breast cancer related deaths. Importantly, YB-1 was a significant factor in a Cox regression model when other variables that typically define risk were taken into consideration. Taking these analyses together with the prognostic value of YB-1 identified in the present study, YB-1 was comparable to tumor size and was superior to HER-2, ER, patient age, or tumor grade as a prognostic marker for RFS and BCSS in the majority of analyses.

During the past few years, several new approaches have been proposed to identify patients at high risk. For example, diagnostic tests have been developed to stratify patients based on gene expression and are currently being prospectively evaluated. The first test, Mammaprint^®^, stratifies node-negative breast cancer patients based on a set of 70 genes using cDNA microarrays [22,23]. A quantitative PCR-based method, Oncotype Dx^®^, is commercially available and appears to define a group of low-risk patients who may be able to avoid chemotherapy [24]. This test was designed to amplify a cassette of 21 genes (16 target genes and five housekeeping genes). Interestingly, within this cassette there are two known YB-1 target genes, namely cyclin B and HER-2. Furthermore, the assay is designed to detect Ki67, which is associated with higher levels of YB-1 [9].

Because YB-1 is expressed in 30% of node-negative breast cancers, based on the data presented herein, it may be useful in stratifying patients thought to be at low risk. We propose that YB-1 could be monitored either using quantitative RT-PCR or in a low-cost manner using immunohistochemistry, just as HER-2 and ER proteins are currently evaluated clinically. We have already optimized the detection of YB-1 using quantitative RT-PCR, as described previously [9], and by immunostaining using an automated system described herein. Thus, this novel biomarker could easily be translated into routine clinical practice for diagnostic purposes. Perhaps YB-1 may serve to define further breast cancer subtypes in general, because we are now beginning to appreciate that triple negative breast cancers can be further divided into five subcategories [18]. Thus, YB-1 can be added to define high-risk patients within the triple negative subtype, as was recently reported for EGFR [25]. Finally, the degree to which the nuclear localization of YB-1 contributes to the aggressiveness of breast cancers continues to be of great interest to us, given its established role as an oncogenic transcription factor. Additional studies will be required, with antibodies that detect nuclear YB-1, to shed light on this.

Although we found that YB-1 is expressed in about 40% of invasive breast carcinomas, the underlying reason for this is unknown. Evidence thus far indicates that YB-1 is not commonly amplified, based on analyses of primary tumors [16] and breast cancer cell lines [26]. There are reports indicating that GATA-1 binds to the YB-1 promoter and suppresses its expression during erythrocyte differentiation [27]. Under stress induced by cisplatin c-myc can also bind to E-boxes located at several sites along the YB-1 promoter [28]. Although stress can induce YB-1, it is still curious to us that breast cancer cell lines express high levels under normal growth conditions, and patients have high levels before chemotherapy or radiation because the tumors from that were under investigation in this report were obtained prior to treatment. To begin to elucidate the regulation of YB-1, we examined its promoter for potential regulatory elements using CONSITE (a predictive algorithm that mitigates against false positives) [29]. Using a 100% stringency criterion, we found that across species the YB-1 promoter has n-myc and snail binding sites [29]. Furthermore, we identified additional regulatory sites on the YB-1 promoter, such as hunchback, rel, sox, and myf [29]. Although n-myc is not thought to play a central role in breast cancer, c-myc does play a role [30], and they both bind to E-boxes. Our virtual screen is consistent with the initial characterization of the YB-1 promoter, in which six E-boxes were identified [31]; this may explain why c-myc reportedly induces its expression in cooperation with p73 [28]. More recently, twist – which also binds to E-boxes – was reported to induce YB-1 [32]. To date, this is the only study addressing the regulation of YB-1 expression specifically in breast cancer.

## Conclusions

The present data provide new impetus to translate these laboratory findings into routine clinical practice, given the remarkable strength of YB-1 as a biomarker for aggressive breast cancer across all subtypes. We anticipate that this new biomarker will create better opportunities to individualize therapy and may ultimately be developed as a novel molecular target.

## Abbreviations

BCSS: breast cancer specific survival; CI: confidence interval; EGFR: epidermal growth factor receptor; ER: estrogen receptor; HER: human epidermal growth factor receptor; HR: hazard ratio; RT-PCR: reverse transcription polymerase chain reaction; PR: progesterone receptor; RFS: relapse free survival; TMA: tissue microarray; YB-1: Y-box binding protein-1.

## Competing interests

The authors declare that they have no competing interests.

## Authors' contributions

SED and GH were responsible for study design, data analysis, and manuscript preparation. DT, HM, TN, and DH were responsible for pathology evaluations. GH, SL, JHL, KG, and MP were responsible for statistical analyses. DH, KG, TN, HM, and DT were responsible for TMA construction and clinical correlates.

## Supplementary Material

Additional file 1 This file shows that YB-1 is strongly associated with poor survival in node-positive and node-negative breast cancers. In node-negative tumors YB-1 was expressed in 33% (572/1,730) of the cases, in which it was strongly associated with reduced BCSS. Similarly, it was expressed in 37% (506/1356) of node-positive breast cancer cases. In these cases YB-1 was also positively associated with reduced BCSS. Click here for file

Additional file 2 This file shows correlations between YB-1 and clinicopathological features of breast cancer. Patients who had tumors expressing YB-1 were younger and tended to have tumors that lacked the ER. There was a positive correlation with increasing tumor grade. Often, tumors that exhibited amplifications in HER-2 also expressed high levels of YB-1. Conversely, YB-1 was not associated with nodal status and weakly related to tumor size. Click here for file

Additional file 3 This file shows that YB-1 is significantly associated with relapse, independent of the type of breast cancer. The expression of YB-1 was associated with shorter RFS (HR = 1.284; *P *= 0.008), independent of breast cancer subtype defined by hormone receptor and HER-2 status, based on a Cox regression analysis. Click here for file

Additional file 4 This file shows that the prognostic significance of YB-1 is associated with reduced BCSS (HR = 1.46, *P *= 6.74 × 10^-7^), independent of tumor subtype. Click here for file

Additional file 5 This file shows a Cox regression model for patients who were treated with surgical resection and no chemotherapy. Nodal status, tumor size, and YB-1 expression were associated with reduced BCSS. YB-1 was better than HER-2 or ER for predicting BCSS. Click here for file

Additional file 6 This file shows a Cox regression analysis for ER-positive patients treated with tamoxifen for 5 years. YB-1 was independently associated with an increased risk for reduced BCSS (HR = 1.703, *P *= 0.022). YB-1 complemented the significance found in node status, tumor size (greater than 2 cm only), and grade. Patient age, small tumors (<2 cm), and HER-2 expression were not independently associated with reduced BCSS. Click here for file
