# Supplementary figures and images for: Redefining prognostic factors for breast cancer: YB-1 is a stronger predictor of relapse and disease-specific survival than estrogen receptor or HER-2 across all tumor subtypes
Source: Breast Cancer Res. 2008 Oct 16;10(5):R86. doi: 10.1186/bcr2156 (PMC2614522; doi:10.1186/bcr2156)

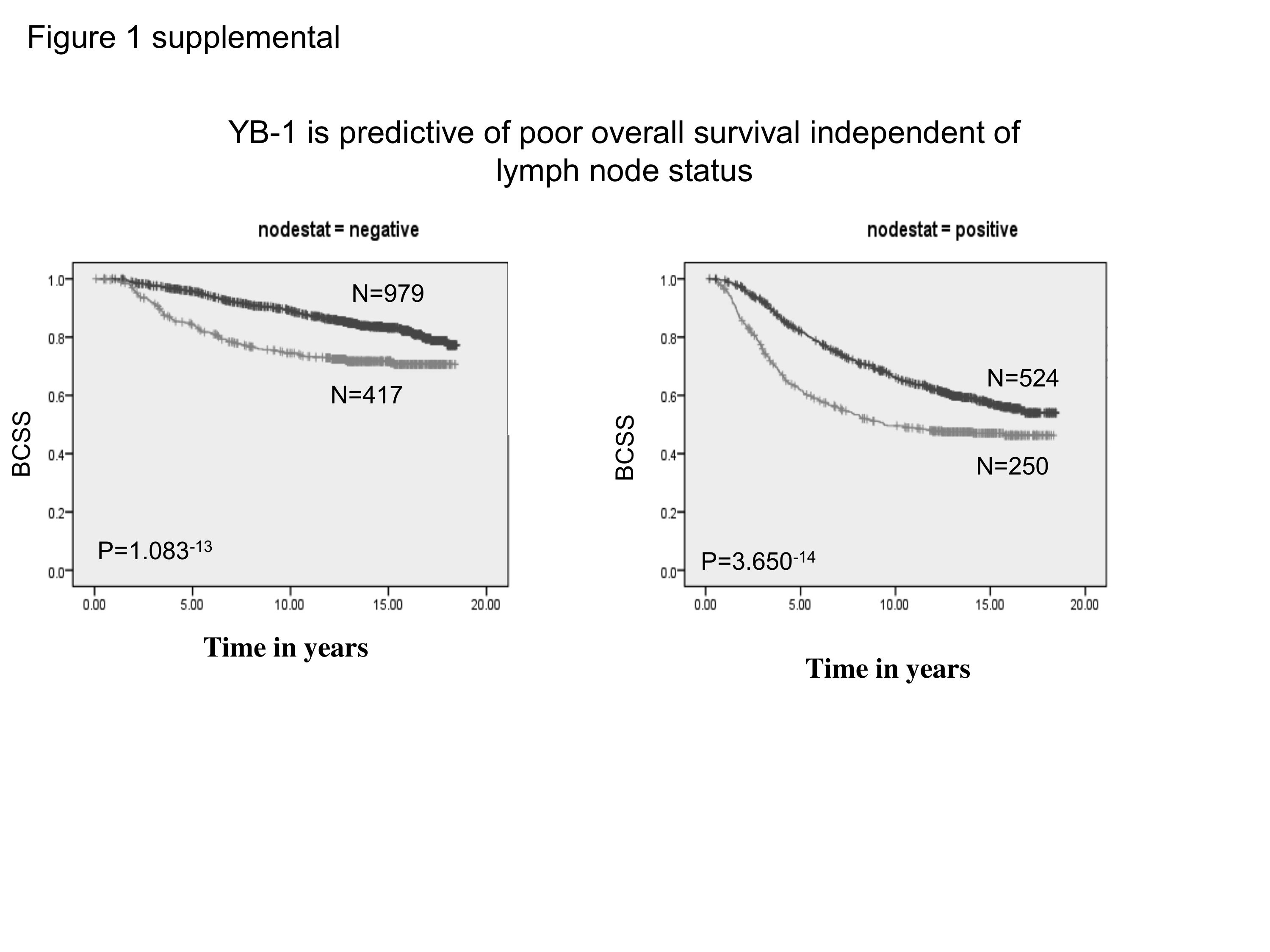

Supplement: Additional file 1 — This file shows that YB-1 is strongly associated with poor survival in node-positive and node-negative breast cancers. In node-negative tumors YB-1 was expressed in 33% (572/1,730) of the cases, in which it was strongly associated with reduced BCSS. Similarly, it was expressed in 37% (506/1356) of node-positive breast cancer cases. In these cases YB-1 was also positively associated with reduced BCSS. [file bcr2156-S1.jpeg]

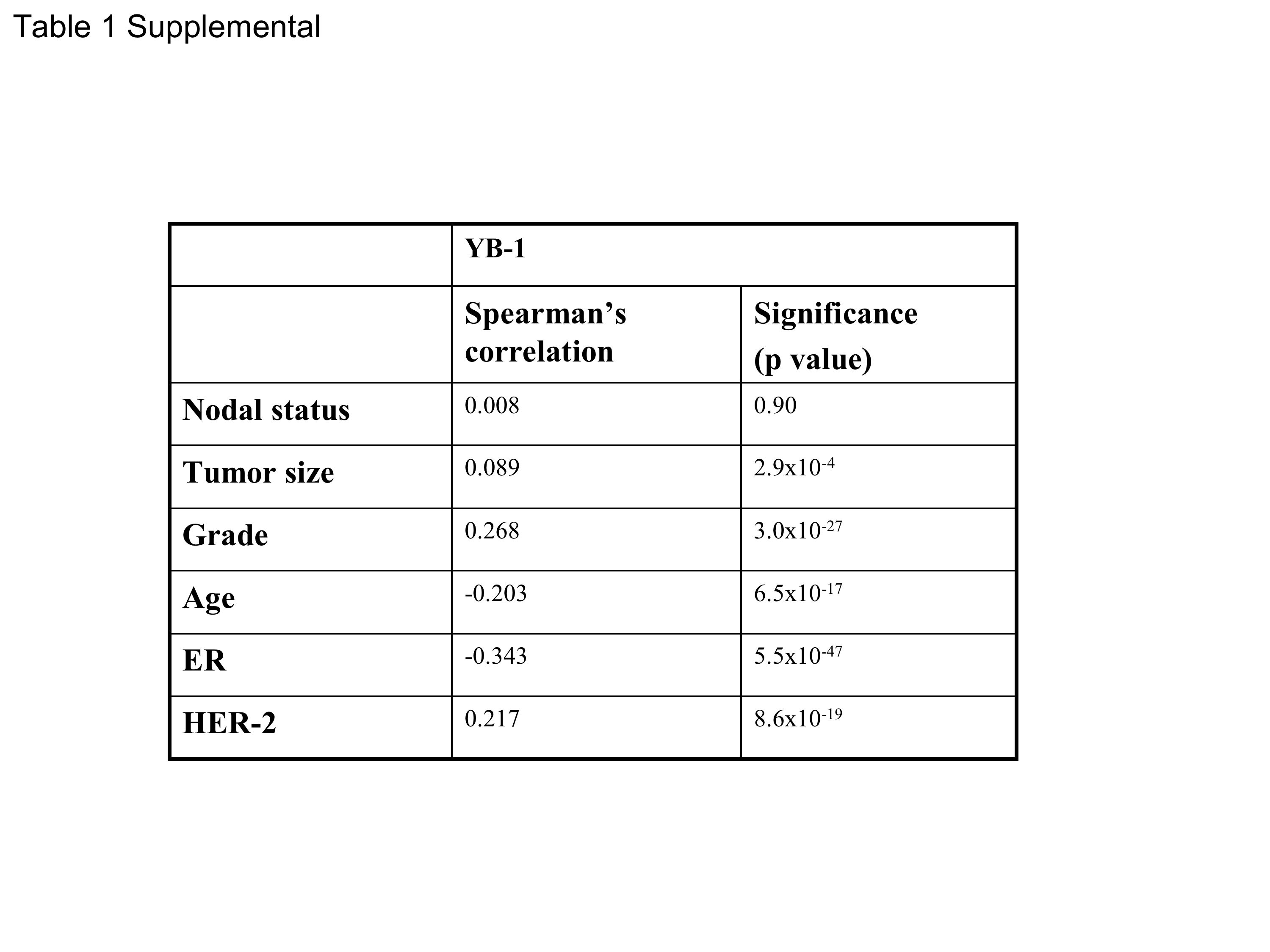

Supplement: Additional file 2 — This file shows correlations between YB-1 and clinicopathological features of breast cancer. Patients who had tumors expressing YB-1 were younger and tended to have tumors that lacked the ER. There was a positive correlation with increasing tumor grade. Often, tumors that exhibited amplifications in HER-2 also expressed high levels of YB-1. Conversely, YB-1 was not associated with nodal status and weakly related to tumor size. [file bcr2156-S2.jpeg]

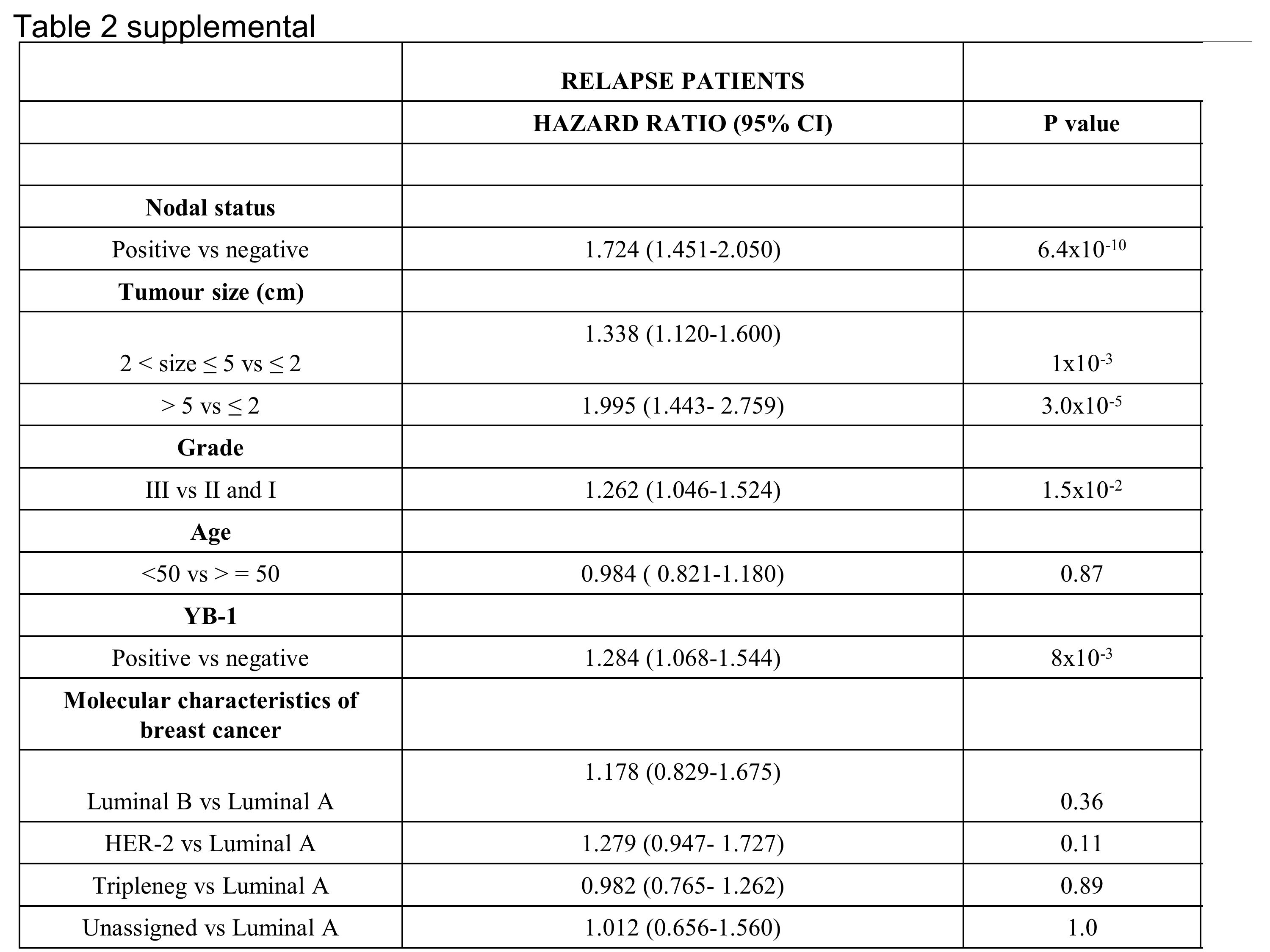

Supplement: Additional file 3 — This file shows that YB-1 is significantly associated with relapse, independent of the type of breast cancer. The expression of YB-1 was associated with shorter RFS (HR = 1.284; P = 0.008), independent of breast cancer subtype defined by hormone receptor and HER-2 status, based on a Cox regression analysis. [file bcr2156-S3.jpeg]

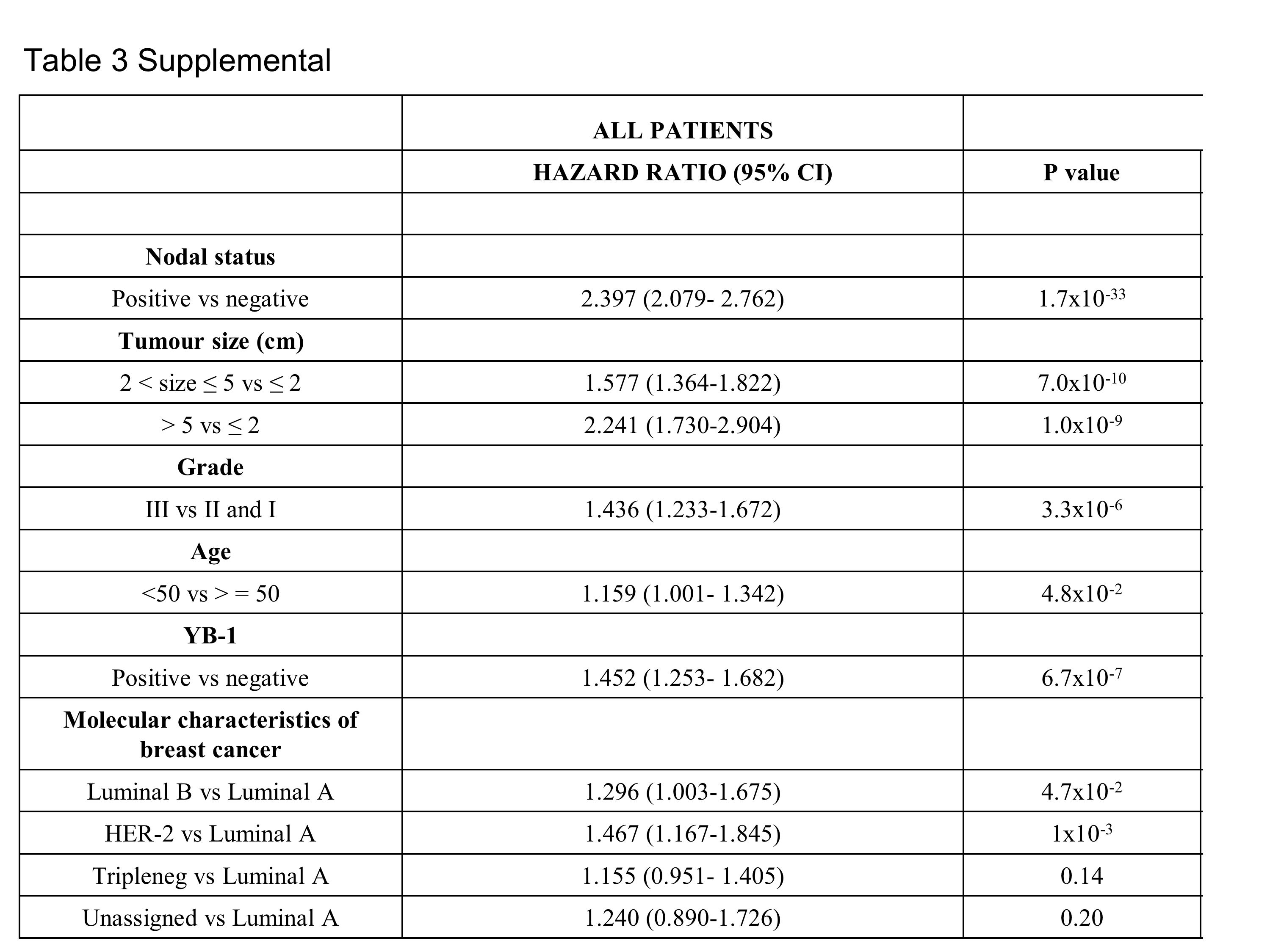

Supplement: Additional file 4 — This file shows that the prognostic significance of YB-1 is associated with reduced BCSS (HR = 1.46, P = 6.74 × 10^-7^), independent of tumor subtype. [file bcr2156-S4.jpeg]

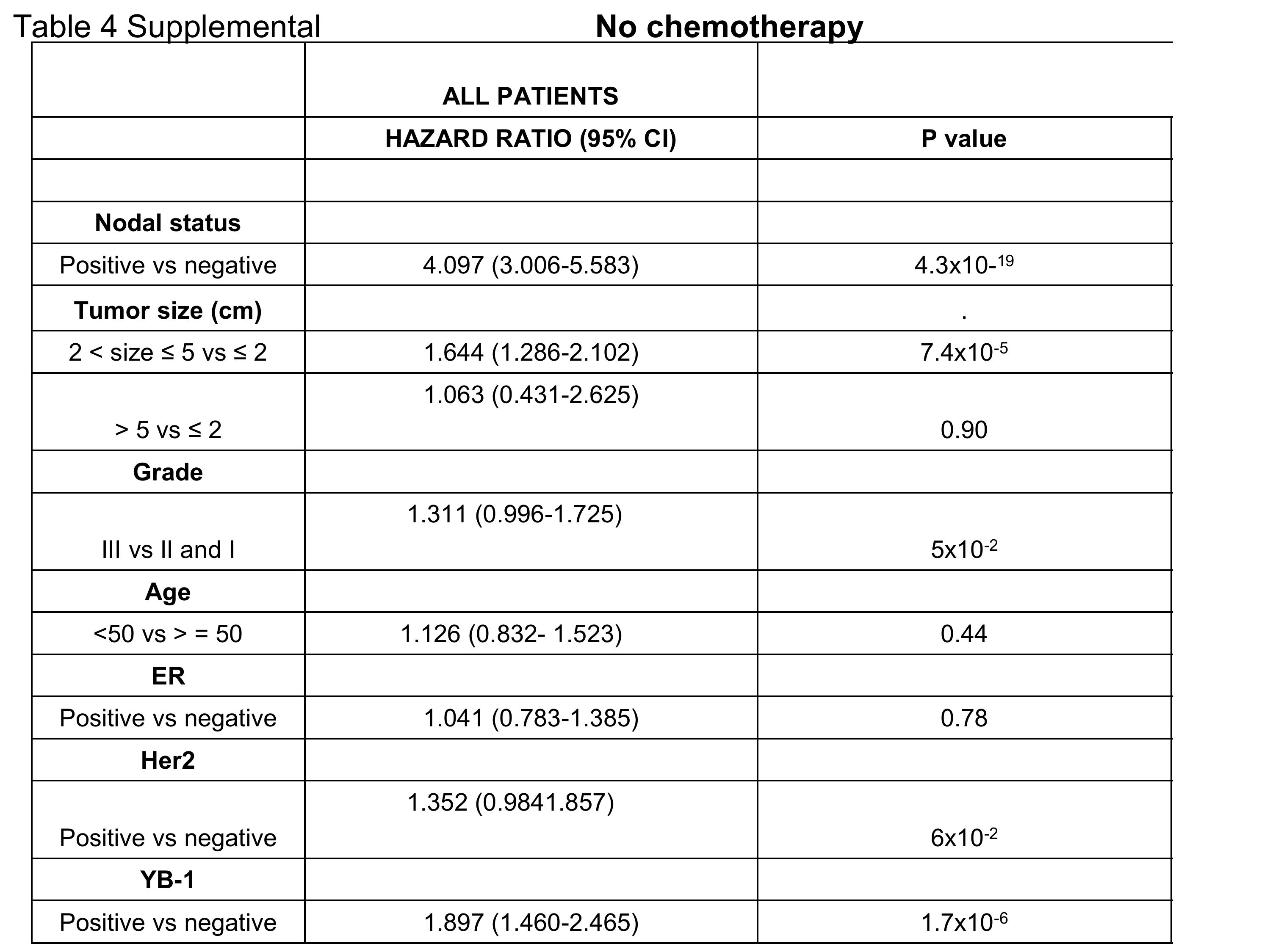

Supplement: Additional file 5 — This file shows a Cox regression model for patients who were treated with surgical resection and no chemotherapy. Nodal status, tumor size, and YB-1 expression were associated with reduced BCSS. YB-1 was better than HER-2 or ER for predicting BCSS. [file bcr2156-S5.jpeg]

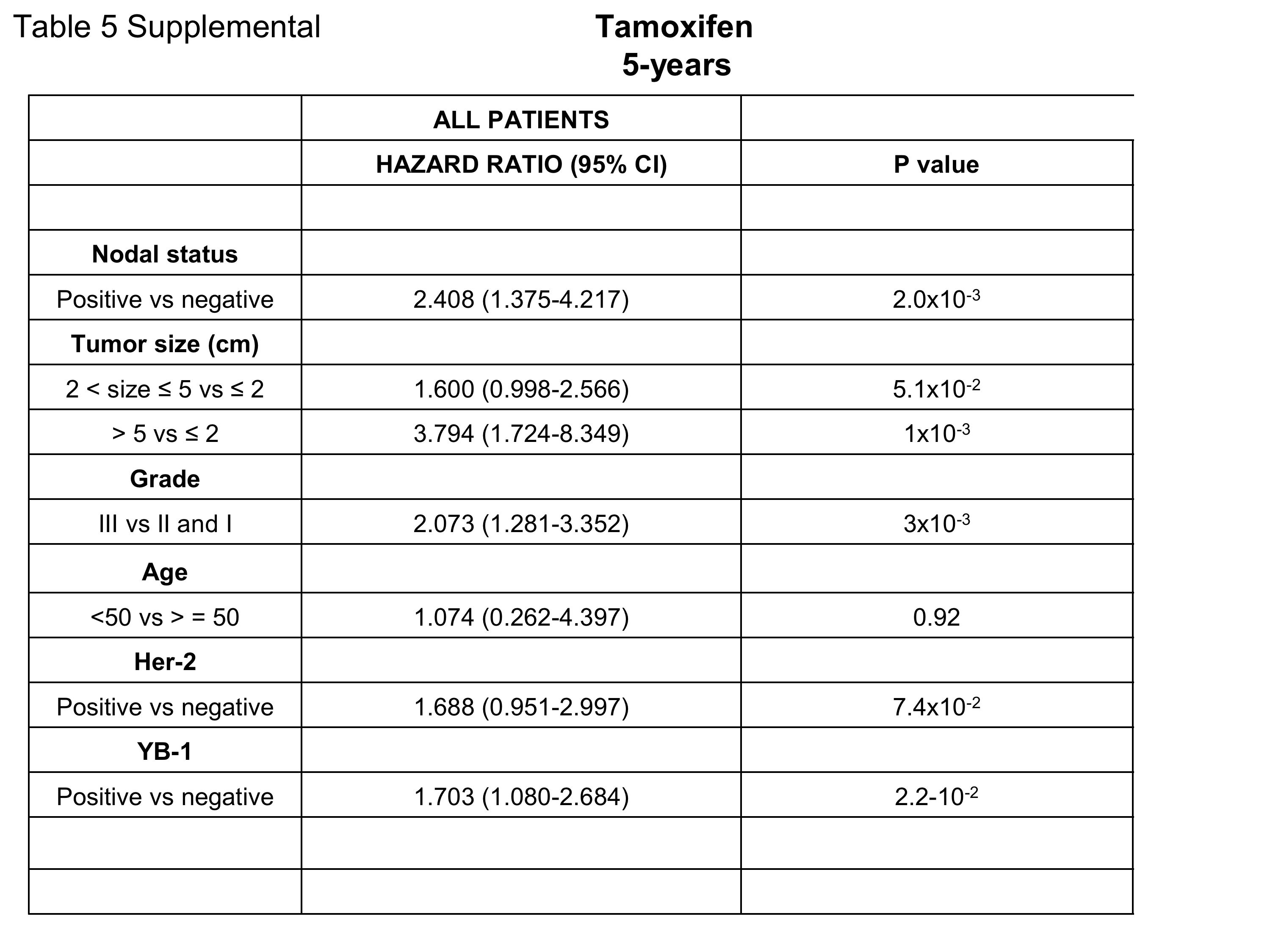

Supplement: Additional file 6 — This file shows a Cox regression analysis for ER-positive patients treated with tamoxifen for 5 years. YB-1 was independently associated with an increased risk for reduced BCSS (HR = 1.703, P = 0.022). YB-1 complemented the significance found in node status, tumor size (greater than 2 cm only), and grade. Patient age, small tumors (<2 cm), and HER-2 expression were not independently associated with reduced BCSS. [file bcr2156-S6.jpeg]
